# Functional coordinates: Modeling interactions between brain regions as points in a function space

**DOI:** 10.1162/netn_a_00264

**Published:** 2022-10-01

**Authors:** Craig Poskanzer, Stefano Anzellotti

**Affiliations:** Department of Psychology, Columbia University, New York City, NY, USA; Department of Psychology and Neuroscience, Boston College, Boston, MA, USA

**Keywords:** Nonlinear, Connectivity, Functional coordinates

## Abstract

Here, we propose a novel technique to investigate nonlinear interactions between brain regions that captures both the strength and type of the functional relationship. Inspired by the field of functional analysis, we propose that the relationship between activity in separate brain areas can be viewed as a point in function space, identified by coordinates along an infinite set of basis functions. Using Hermite polynomials as bases, we estimate a subset of these values that serve as “functional coordinates,” characterizing the interaction between BOLD activity across brain areas. We provide a proof of the convergence of the estimates in the limit, and we validate the method with simulations in which the ground truth is known, additionally showing that functional coordinates detect statistical dependence even when correlations (“functional connectivity”) approach zero. We then use functional coordinates to examine neural interactions with a chosen seed region: the fusiform face area (FFA). Using *k*-means clustering across each voxel’s functional coordinates, we illustrate that adding nonlinear basis functions allows for the discrimination of interregional interactions that are otherwise grouped together when using only linear dependence. Finally, we show that regions in V5 and medial occipital and temporal lobes exhibit significant nonlinear interactions with the FFA.

## Author Summary

In this paper, we introduce a new method to investigate not only whether a set of brain areas interact, but also *how* the activity in those regions is related. To do this, we model the functional relationships between activity in distinct brain areas as points in a function space that can be described by “functional coordinates” along multiple basis functions. First, we demonstrate the efficacy of this novel method on simulated data; next, we apply it to real neural data, reporting evidence of nonlinear interactions. Functional coordinates can serve as a tool in future studies to further our understanding of the complex interactions across the brain.

## INTRODUCTION

The use of linear and nonlinear models for the analysis of neuroimaging data is at the center of a lively debate (Ivanova et al., 2021). On one hand, proponents of linear models argue that nonlinear models can lead to overfitting issues given the amount of data that is typically available (Misaki et al., 2010). Linear models have been found empirically to yield insights about the brain when used for pattern classification (Anzellotti, Fairhall, & Caramazza, 2014), representational similarity analysis (Kriegeskorte, Mur, & Bandettini, 2008), and functional connectivity (Biswal et al., 1995). On the other hand, proponents of nonlinear models argue that linear models are not biologically plausible: firing rates of single neurons are integrated nonlinearly within dendrites (Beniaguev, Segev, & London, 2021; Xu et al., 2012), and nonlinear transformations are essential to perform many of the tasks humans need to solve. Thus, while linear models might be effective to test whether a brain region encodes a given set of features, they might fall short of capturing interactions between brain regions with a complexity sufficient to enable the understanding of cognitively relevant computations.

More broadly, research on the statistical dependence between the responses in different regions (“functional connectivity”) has focused on studying *whether* given pairs of brain regions interact; however, there is a need for methods that can be used to investigate *how* they interact—to distinguish between different kinds of mappings that transform information from brain region to brain region. Even interactions between brain regions displaying similar strengths of functional connectivity could belie very different nonlinear computations.

A recurring criticism of nonlinear models is based on the difficulty to interpret them. In decoding analyses, linear models make it easier to distinguish the contribution of neural information processing up to the brain region whose responses are being measured from the contribution of the decoder applied to extract information from that brain region (see Kamitani & Tong, 2005; Norman et al., 2006). By contrast, nonlinear decoders can transform the neural responses they receive as inputs to an extent that might lead to ambiguity about the nature of representations in the brain region that is being investigated. To illustrate this point with an example, if we could use any nonlinear decoder, and we had noiseless data from every single neuron in early visual cortex, we should be able to use these data to perform view-invariant object classification. After all, the brain itself can perform view-invariant object classification using early visual cortex responses as input. However, this finding would not support the conclusion that early visual cortex encodes view-invariant representations of objects, because the nonlinearities in the decoder would have likely been necessary for view invariance to occur.

This criticism of nonlinear decoders is largely motivated by the “standard” analysis strategy used in the literature. This standard strategy consists of training one model to achieve the highest possible decoding accuracy, given the responses from a brain region as input, and interpreting decoding with significantly-above-change accuracy as evidence that the brain region encodes information about the property that was successfully decoded (Anzellotti et al., 2014). Similarly, in the field of functional connectivity, the best estimate of the statistical dependence between the responses in two different regions is calculated, and significance is interpreted as evidence for the dependence between those regions’ responses (Anzellotti, Caramazza, & Saxe, 2017a; Greicius et al., 2003).

In this article, we introduce a new perspective. We suggest that nonlinear models should not be used to replace linear models—instead, information about the relative contributions of linear and nonlinear models should be preserved. Rather than selecting a single model and using its performance to determine the strength of the interaction between two regions, we propose to use a family of models and to treat the respective contributions of different models as a set of “functional coordinates” that characterizes not just the strength, but also the type of interaction between regions. From a mathematical perspective, the proposed approach is rooted in seeing the problem of connectivity as function estimation, and it is inspired by the idea that a function can be expressed as a point in a Hilbert space, having as coordinates its projections on a (infinite) set of basis functions.

Previous research has introduced nonlinear approaches to the study of connectivity using Dynamic Causal Modeling (DCM; Stephan et al., 2008), Granger causality (Marinazzo, Pellicoro, & Stramaglia, 2008), mutual information (Lizier et al., 2011), and Multivariate Pattern Dependence (MVPD; Anzellotti et al., 2017b). However, by and large these methods have followed the traditional approach of building one model that performs as accurately as possible and interpreting the quality of fit, parameter values, or accuracy as evidence for the existence of interactions. The approach we propose in this article, instead, focuses on distinguishing between different kinds of interactions between regions, offering a new technique that can reveal differences even between region pairs whose overall correlations or statistical dependencies are comparable in strength.

## METHODS

**Figure 1.**
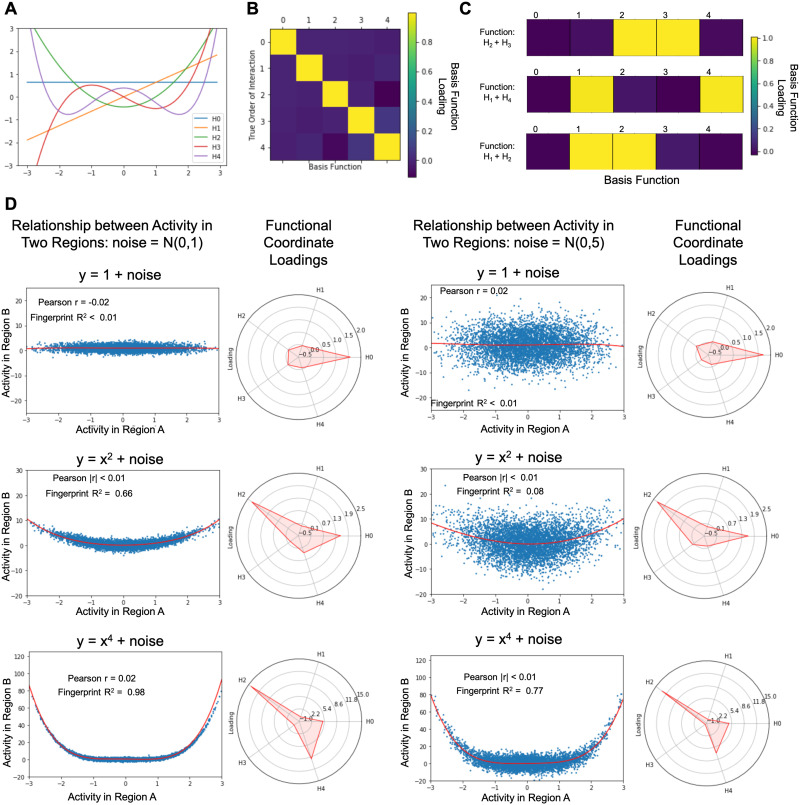
Validation with synthetic data. (A) The first five Hermite polynomials. (B) Functional coordinates identify the underlying relationship between two sets of simulated data. We generated five sets of *x*-*y* pairs where the true relationship between each subsequent pair of *x* and *y* is one of the first five Hermite polynomials (ex. *y*_1_ = *H*_1_(*x*_1_)). As expected, the coordinate estimates assign coefficients near 1 to the polynomial in the basis set that defines the relationship between *x* and *y*, and coefficients near 0 to all others. (C) When the simulated data are generated using a linear combination of two basis functions (ex. *y*_1_ = *H*_1_(*x*_1_) + *H*_2_(*x*_1_)), the resulting functional coordinates reveal loadings near 1 for each contributing basis function, and loadings near zero for all other basis functions. (D) Nonlinear coordinates can approximate U-shaped interactions that would be indistinguishable using standard correlation analysis. We generated synthetic data where the relationship between activity in Region A and activity in Region B have a symmetric relationship (*y* = 1 + *noise*, *y* = *x*^2^ + *noise*, and *y* = *x*^4^ + *noise*). A range of noise was simulated by drawing from two normal distributions with mean 0 and standard deviation 1 and 5, respectively. The estimated function is plotted in red and the loadings on the functional coordinates are illustrated in radar plots alongside each graph.

The study of univariate statistical dependence between pairs of brain regions offers an ideal test case for the use of functional coordinates. The univariate nature of the problem prevents a combinatorial growth in the number of nonlinear terms, and the continuous (rather than discrete) nature of the outputs makes it possible to use a simple basis set such as Hermite polynomials (Hermite, 1864; Szeg, 1939). We used Hermite polynomials in this work as they are defined on all ℝ and have a natural multivariate extension, but the same logic can be applied to other basis sets such as Fourier basis functions or Legendre polynomials. For additional convenience, in this study we divide each Hermite polynomial of order *n* by $\sqrt{\sqrt{2\pi }n!}$ to render them an orthonormal basis (see Van Eijndhoven & Meyers, 1990; Supporting Information, The first five normalized Hermite polynomials; see also Figure 1A).

### Theory

#### Expressing functions with a truncated orthonormal Hermite basis.

We will consider the average response in a predictor region at time *t* (which we will denote with *x*_*t*_) and the average response in a target region also at time *t* (which we will denote with *y*_*t*_). Modeling the dependence between *x*_*t*_ and *y*_*t*_ as *y*_*t*_ = *f*(*x*_*t*_) + *ϵ*_*t*_, we will aim to characterize the function *f* that minimizes the error *E*(*f*) = ∑_*t*_ $\epsilon_{t}^{2}$. For convenience (and without loss of generality), we will normalize the inputs and outputs to have mean 0 and standard deviation 1. We will make the assumption that the function *f* is in the Hilbert space of functions from the interval ℝ to ℝ satisfying

$$\int_{-\infty }^{\infty }{\left|f\left(x\right)\right|}^{2}p\left(x\right)dx<\infty ,$$ (1)

where

$$p\left(x\right)=\frac{1}{\sqrt{2\pi }}e^{-\frac{1}{2}x^{2}}.$$ (2)

This is a large space of functions, and it should be sufficient to approximate well the relationship between the predictors and the targets of prediction.

The inner product between any two functions *g*_1_, *g*_2_ defined as

$$\langle g_{1},g_{2}\rangle =\int_{-\infty }^{+\infty }g_{1}\left(x\right)g_{2}\left(x\right)p\left(x\right)dx.$$ (3)

Hermite polynomials are a basis set of the Hilbert space. If we knew the function *f* that minimizes the error *E*(*f*), we could express it as an infinite set of coordinates *c*_*i*_ such that

$$f\left(x\right)=\sum_{i=0}^{\infty }c_{i}h_{i}\left(x\right),$$ (4)

where *h*_*i*_ is the *i*th Hermite polynomial (normalized to have norm 1). Thus

$$c_{i}=\int_{-\infty }^{+\infty }f\left(x\right)h_{i}\left(x\right)p\left(x\right)dx\;\forall \;i\in \mathbb{N},$$ (5)

To make this strategy applicable in practice, we need to address two challenges. First, we cannot calculate an infinite number of coordinates; therefore, we will truncate Hermite polynomials to a specified order. The optimal order at which to truncate the polynomials can depend on the amount of data and the nature of the interactions between the regions studied. In the present article, the main focus is not to determine the optimal number of polynomials. Therefore, we will use polynomials up to the fifth order (future studies can use variance explained in independent data as a metric for the selection of the number of polynomials). Second, we do not know the function *f*, we only have a training dataset containing pairs of observations (*x*_1_, *y*_1_), …, (*x*_*T*_, *y*_*T*_). To address this second challenge, for each Hermite polynomial *h*_*i*_ we will estimate the corresponding coordinate as

$${\hat{c}}_{i}=\text{argmin}\left(\sum_{t=1}^{T}{\left(y_{t}-c_{i}h_{i}\left(x_{t}\right)\right)}^{2}\right).$$ (6)

The coordinates $\hat{c}$_*i*_ approximately characterize the function *f* (up to the precision afforded by the truncation).

#### Convergence of the coordinate estimates.

If $\hat{c}$_*i*_ is a “good” estimate of *c*_*i*_, as the number of observations grows the estimate should converge to the true value *c*_*i*_. We demonstrate this property in the following Lemma.

We will assume that the data-generating process is approximately Normal, and since we normalize our input data to have mean *μ* = 0 and standard deviation *σ* = 1 we have approximately that *x* ∼ 𝒩(0, 1) (see Supporting Information, Normality of the Data; Figure S7). Since the error function is convex, we can calculate $\hat{c}$_*i*_ by setting

$$\frac{d}{{dc}_{i}}\sum_{t=1}^{T}{\left(y_{t}-c_{i}h_{i}\left(x_{t}\right)\right)}^{2}=0$$ (7)

which yields

$${\hat{c}}_{i}=\frac{\sum_{t=1}^{T}y_{t}h_{i}\left(x_{t}\right)}{\sum_{t=1}^{T}h_{i}{\left(x_{t}\right)}^{2}}=\frac{\sum_{t=1}^{T}f\left(x_{t}\right)h_{i}\left(x_{t}\right)}{\sum_{t=1}^{T}h_{i}{\left(x_{t}\right)}^{2}}.$$ (8)

As the number of observations increases, taking into account the fact that *x* ∼ 𝒩(0, 1), we have that

$$\lim_{T\to \infty }\frac{\sum_{t=1}^{T}f\left(x_{t}\right)h_{i}\left(x_{t}\right)}{\sum_{t=1}^{T}h_{i}{\left(x_{t}\right)}^{2}}=\frac{\int_{-\infty }^{+\infty }f\left(x\right)h_{i}\left(x\right)p\left(x\right)dx}{\int_{-\infty }^{+\infty }h_{i}{\left(x\right)}^{2}p\left(x\right)dx}$$ (9)

and since we have used normalized Hermite polynomials that form an orthonormal basis,

$$\int_{-\infty }^{+\infty }h_{i}{\left(x\right)}^{2}p\left(x\right)dx=1.$$ (10)

In conclusion,

$$\lim_{T\to \infty }{\hat{c}}_{i}=\int_{-\infty }^{+\infty }f\left(x\right)h_{i}\left(x\right)p\left(x\right)dx=c_{i}.$$ (11)

Note that based on this observation, in the presence of nonnormally distributed data, it might be possible to estimate the probability density of the data *q*(*x*) and develop a basis set of polynomials that are orthonormal with respect to the inner product defined by

$$\langle g_{1},g_{2}\rangle =\int g_{1}\left(x\right)g_{2}\left(x\right)q\left(x\right)dx$$ (12)

where the integral is computed over the domain of *q*.

### Application

#### Generating synthetic data.

In order to validate the performance of the functional coordinates on data in which the true relationship between datasets is known, we generated synthetic data in which we could define normally distributed vectors of *x* (mean = 0, standard deviation = 1). Next, we created a vector of *y* values such that

$$y=f\left(x\right),$$ (13)

where *f* is the explicit relationship that we will seek to approximate using functional coordinates.

#### Participants and stimuli.

All data used in this study were made publicly available as part of the *StudyForrest* dataset (Hanke et al., 2016). The study procedures were approved by the Ethics Committee of Otto-von-Guericke University, and all participants provided informed consent (for more details, see Hanke et al., 2016). These data consist of fMRI scans of 15 subjects (6 female, ages 21–39, mean = 29.4) as they watched the movie *Forrest Gump*. Data from one subject were removed from the analyses after a technical error caused a failure in the fMRIPrep preprocessing procedure in multiple attempts (see also Li, Saxe, & Anzellotti, 2019).

After providing consent, participants watched the movie in the scanner over the course of eight functional runs (approximately 15 minutes each). Additionally, subjects performed a localizer task incorporating 24 grayscale images from each of the following six categories: faces, bodies (without heads), small objects, houses, outdoor scenes of nature and streets, and phase scrambled images; for more information about the localizer task, please see Sengupta et al. (2016).

All scans were performed in a 3T Philips Achieva dStream MRI scanner with a 32-channel head coil. BOLD responses were recorded at 3 × 3 × 3 mm resolution with T2*-weighted echo-planar (2 sec-repetition time (TR)) imaging sequence. See Hanke et al. (2016) for more details on image acquisition.

#### Data preprocessing.

Data were preprocessed according to the fMRIPrep pipeline described in Esteban et al. (2019). This procedure combines the following steps: T1-weighted anatomical images were smoothed and skull-stripped using advanced normalization tools; brain images were segmented into white matter (WM), gray matter, and cerebrospinal fluid (CSF) using FSL-FAST (Zhang, Brady, & Smith, 2001); FSL-MCFLIRT (Jenkinson et al., 2002) was used to correct functional scans for head movement; functional scans were aligned with the corresponding anatomical image using boundary-based coregistration implemented in FSL-FLIRT.

Connectivity analyses are particularly susceptible to fluctuations in the BOLD signal as the result of motion and respiration; in order to effectively examine the interactions between the activity across regions, it is necessary to incorporate denoising approaches that take measures to remove signal of no interest. To this end, after preprocessing, the data were additionally denoised using CompCor (Behzadi et al., 2007). In this method, noise is removed from the functional data by regressing out the first five principal components extracted from the combined WM and CSF data. The central assumption of CompCor is that the signal extracted from the WM and CSF is uninformative, and thus by using these anatomically defined sources of nonneural signal, one can predict and remove fluctuations in the neural data that are of no interest. Although this method may not be able to perfectly distinguish the signal of interest from noise, it has been shown to effectively remove spurious signal in studies examining multivariate and nonlinear interactions (Li et al., 2019; Poskanzer et al., 2022; Power et al., 2012).

#### Localization of the region of interest.

To define the region of interest (ROI), data from the initial run of the functional localizer task were modeled using a standard GLM in FEAT (Woolrich et al., 2001), which included each object category from the task as a predictor. All predictors were convolved with a gamma hemodynamic response function. The fusiform face area (FFA) was located among the regions maximizing the contrast in activity for faces compared with all other stimuli. Next, we selected the single voxel within the FFA with the maximum *t*-value for the contrast of faces over all other categories. We then created a 9-mm sphere surrounding this voxel and selected the 80 voxels within this sphere with the largest *t*-values for faces > nonfaces. These 80 voxels served as the FFA ROI for our analyses. This procedure has additionally been reported in Fang et al. (2019) and Poskanzer et al. (2022).

#### Data analysis.

Let’s consider an fMRI dataset, and a seed region. In this study, we used the FFA as the seed region. We then used functional coordinates to characterize the relationship between the normalized responses in FFA and the normalized responses in each other voxel *v* in gray matter. For each participant, and for each voxel in gray matter (normalized to MNI space), we applied the method described in the previous section using as *x*_*t*_ the average response in FFA at time *t*, and as *y*_*t*_ the response in the voxel *v* at time *t*. This procedure yielded a five-dimensional vector of the estimated coordinates $\hat{c}$_1_, …, $\hat{c}$_5_ along the first five Hermite polynomials. Next, we used *k*-means clustering with the Akaike information criterion (AIC) to identify clusters of voxels using the five-dimensional vectors from all participants. This approach identified the optimal number of clusters from the data by balancing complexity and quality of fit. Each cluster corresponded to a distinct kind of nonlinear interaction between brain regions. Finally, clustering was visualized by color coding each voxel in gray matter by the cluster to which it is assigned most often, with saturation increasing as a function of the proportion of participants for whom the voxel was assigned to the most frequent cluster.

It is important to note that because functional coordinates describe the function that transforms BOLD responses in region A into responses in region B, it is necessary for a researcher interested in the relationship between two brain areas to select which area is region A (the predictor) and which is region B (the target of prediction). In the case in which a researcher does not have an a priori hypothesis about direction of the relationship between these brain areas, it is recommended to calculate functional coordinates to estimate the relationship in both directions.

To test our model’s ability to distinguish between voxels based on their nonlinear interactions with the FFA, we compared clustering solutions for the five-dimensional functional coordinates with the optimal clustering solution across the loadings for only the linear basis vector. In this way, we were able to determine the subsets of voxels with similar linear loadings that were differentiated by their nonlinear components. This analysis clusters voxels according to the *type* of interaction between the voxel’s activity and the activity in the seed region, thus highlighting brain areas with distinct functional relationships to the FFA. Finally, using Statistical Non-Parametric Mapping (SnPM; SnPM, 2013), which uses permutation tests in order to determine the significance (*p* values), we tested whether the magnitude of the nonlinear basis vector loadings for all voxels (using a cluster forming threshold of 0.0001) were significantly nonzero to determine where neural interactions with the FFA were significantly nonlinear in nature.

Testing statistical significance for each of the higher order coordinates requires controlling for multiple comparisons. We suggest two possible approaches to mitigate the challenges associated with multiple comparison correction. A first approach consists in computing the explained variance for groups of loadings of interest. For example, the contribution of nonlinear components could be tested by comparing the explained variance including Hermite polynomials of order greater than 1 to the explained variance using polynomials of order up to 1. This approach could be also used to test other questions, for instance, it could be used to investigate the contribution of polynomials with even order to that of polynomials with odd order to evaluate the extent to which the response of the target region is symmetrical around the average response of the seed region. Symmetrical effects could capture interpretable relationships between brain areas—such as cases in which the responses in one region might be driven by both increases and decreases in the response in another region compared to baseline.

A second approach entails using part of the data as a functional localizer. For example, one experimental run could be used to identify particular polynomial orders that show effects of interest. Then, independent data could be used to test specifically the significance of the effects for those polynomial orders, much like regions of interest are used to tackle multiple comparison issues in the spatial domain.

## RESULTS

### Estimated Coordinates Match the Ground Truth in Simulated Data

In order to test the efficacy of our novel analysis to detect the functional relationship between two patterns of activity, we used simulated data to model a series of potential interactions between generated seed-target datasets. By manipulating the function used to create target data from a set of simulated seed data, we can test the ability of the functional coordinate analysis to correctly model the selected relationship. Our results illustrate that through estimating loadings on the first five basis vectors of our selected functional space, we can accurately characterize the generative function of the target data for the functions tested (see Figure 1; more complex functions might require a higher number of polynomials).

#### Individual polynomials.

Starting with a seed sample of 10,000 normally distributed data points (mean = 0, *SD* = 1), we defined five sets of target data as *H*_1–5_(*seed*) where *H*_1–5_ represents each of the first five normalized Hermite polynomials. Given that the interactions between the seed and target data were selected to be the five basis vectors by which we are measuring functional space, if our analysis correctly identifies the underlying computation that generates the target data, we would expect to see a loading of 1 on the relevant basis vector and loadings of 0 on all other basis vectors. For each set of target data, we found a five-dimensional set of functional coordinates with a loading of 1.00 for the basis vector governing the underlying relationship between the seed and target data, as well as loadings with an absolute value < 0.12 for all other basis vectors (see Figure 1B).

#### Combinations of polynomials.

To further probe the ability of functional coordinates to capture more complex relationships between seed and target data, we next generated target data using a linear combination of multiple Hermite polynomials. Assuming the underlying function that describes the interaction between the seed and target data is an unweighted combination of a subset of Hermite polynomials, the resulting functional coordinates should consist of a vector of loadings with values of 1 for the given subset of polynomials and loadings of 0 for all other basis vectors. In our validation, we demonstrate in three cases (*H*_2_ + *H*_3_, *H*_1_ + *H*_4_, and *H*_1_ + *H*_2_) that these functional coordinates capture the selective loadings on the relevant basis vectors (see Figure 1C).

### Nonlinear Coordinates Can Capture U-Shaped Dependencies

One key benefit of characterizing functional interactions using nonlinear functional coordinates is that they offer considerably more explanatory power than a standard Pearson correlation. One illustrative example in which our functional coordinate analysis outperforms correlation tests arises in the instance when interactions are governed by a symmetrical underlying function. In the case of a U-shaped relationship, the magnitude of activity in a seed region, either negative or positive, results in a proportional positive response in the target ROI. This type of relationship could be particularly useful to understand brain regions that might show greater sensitivity to the magnitude of the deviation from baseline of the responses in another brain region, as opposed to its direction (positive/negative).

Importantly, testing for these patterns of related activity using Pearson’s correlation will result in null findings for any significant interactions between the symmetrical data. Because Pearson’s “r” is a measure of the *linear* correlation of variables, it is not especially informative when seeking to explore *nonlinear* relationships between sets of data. In contrast to the inability of correlation coefficients to distinguish between null relationships and U-shaped dependencies, estimating a functional coordinate to map interactions provides a much more informative model of any symmetrical dependencies (see Figure 1D).

In our simulated experiments, we show that not only do functional coordinate estimations tightly track the shape of nonlinear functions (e.g., *x*^2^ and *x*^4^), but also, that these functional coordinates are able to differentiate between dependencies that would otherwise be indistinguishable using measures of linear correlation (see Figure 1D). To highlight the explanatory power of our functional coordinate analysis, we generated three sample dependencies where Pearson r ≈ 0: *y* = 1 + *ε*, *y* = *x*^2^ + *ε*, and *y* = *x*^4^ + *ε*, where *ε* represents a random amount of noise. In each case, *x* was defined as a normally distributed vector of 5,000 values (*mean* = 0, *SD* = 1). In order to demonstrate the robustness of functional coordinates we used simulated two distinct sets of noise by selecting 5,000 values from a normal distribution *mean* = 0, *SD* = 1, and *mean* = 0, *SD* = 5. Importantly, while all three of these interactions show |*r*| <= 0.02, each relationship is described by a unique functional coordinate: for noise with standard deviation of 1 (*y* = 1: [1.56, −0.02, −0.01, 0.02, −0.05]; *y* = *x*^2^: [1.51, 0.00, 2.26, −0.03, 0.36]; *y* = *x*^4^: [5.05, 0.21, 14.26, 0.62, 9.06]) and for noise with standard deviation = 5 (*y* = 1: [1.63, 0.25, 0.07, 0.13, 0.10]; *y* = *x*^2^: [1.59, −0.03, 2.23, 0.19, 0.01]; *y* = *x*^4^: [4.71, 0.08, 13.07, −0.56, 6.99]). In this way, these functional coordinates allow for the identification and modeling of U-shaped dependencies that could otherwise be overlooked in a standard correlation analysis.

### Nonlinear Coordinates Identify More Clusters Than Linear Coordinates

**Figure 2.**
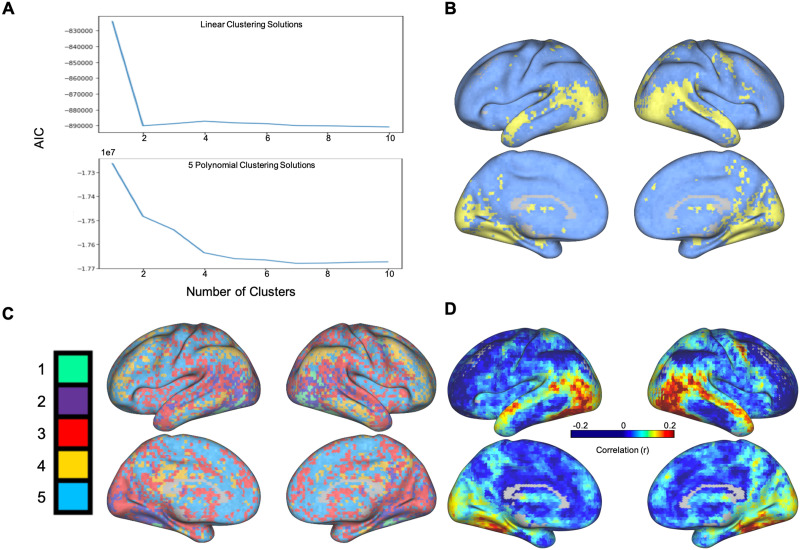
(A) Using *k*-means clustering, as the number of clusters (*k* value) increases, the Akaike information criterion (AIC) values decrease. The optimal clustering solution occurs when the AIC stops substantially decreasing. Top: Linear functional coordinates (Hermite polynomials of degree 1). The elbow occurs at *k* = 2 clusters, meaning the optimal number of clusters across linear coordinates is 2. Bottom: Nonlinear functional coordinates (Hermite polynomials of degree 0 through 4). The elbow occurs at *k* = 5 clusters. (B) Visualization of the cluster assignments of different voxels based on the linear functional coordinates. For each voxel, we used the mode cluster assignment across subjects to determine that voxel’s final cluster value. Each cluster is represented by a different color, and the intensity of the color represents the percentage of subjects sharing that voxel’s cluster assignment (lighter colors denote a higher percentage). (C) Visualization of the cluster assignments of different voxels based on the nonlinear functional coordinates (generated with the same approach described in panel B). There was no difference in the clustering solution using all five basis vectors and when excluding the 0th-order basis vector. (D) Functional connectivity was calculated between the FFA and each individual voxel for all subjects. After averaging the correlation values across all subjects, we plotted the resulting average correlation values across the cortex. The pattern of functional connectivity with the FFA illustrates the highest correlations with regions spanning the ventral, and dorsal temporal lobe and much of the visual cortex. These results parallel clustering solution using linear coordinates.

Exploring neural data using functional coordinates provides unique insight into the *types* of interactions between brain regions. Moreover, using additional, nonlinear basis vectors to estimate interregional dependencies allows for a heightened sensitivity to more complex interactions. Other approaches like mutual information can be used to capture nonlinear dependence between brain regions (Lizier et al., 2011; Wang et al., 2015). However, functional coordinates are unique in that they do not characterize the interaction between two brain regions using a single value that reflects the strength of the dependence; instead, functional coordinates characterize the interaction between two regions with a multidimensional vector encoding the contributions of different Hermite basis functions. We used these multidimensional vectors to subdivide cortex into distinct clusters of voxels with different types of interactions with the FFA. To quantify the advantage of using multidimensional functional coordinates, we compared the optimal clustering solutions for voxels across participants using five-dimensional functional coordinates (leveraging the first five normalized Hermite polynomials as basis vectors) and one-dimensional functional coordinates (using only the first order, linear polynomial). In both cases, we calculated the AIC for clustering solutions ranging from 1 to 10 clusters. In order to counterbalance the increased explained variance of more clusters with the potential to overfit the data with too many clusters, the optimal number of clusters is determined by locating the “elbow” of the plotted AIC values—where an increase in the number of clusters no longer corresponds with a substantial decrease in information lost. Importantly, for the linear functional coordinate analysis, the optimal number of *k*-clusters was found at *k* = 2, while for the nonlinear functional coordinate analysis, the best solution existed at *k* = 5 (see Figure 2A and C).

After determining the ideal *k* value for the linear and nonlinear analyses, we used *k*-means to group voxels by their linear coordinates and nonlinear coordinates across all subjects (see Figure 2B and C). Our analysis of the linear coordinates yielded two distinct clusters across the brain. The smaller (yellow in Figure 2B) of the two clusters encompasses the dorsal and ventral temporal lobes bilaterally, as well as large sections of the bilateral visual cortex. The larger cluster (blue in Figure 2B), comparatively, sprawls across the frontal and parietal lobes, as well as the lateral temporal lobes in both hemispheres. In contrast, the five clusters generated from the nonlinear coordinates show a unique division of the cerebral cortex (Figure 2C). The green cluster is concentrated around a series of face selective regions, including the FFA (our seed region), the superior temporal sulcus, and the occipital face area. The purple cluster covers the majority of the visual cortex as well as the lateral and ventral temporal lobes. The red cluster is more distributed, encompassing bilateral sections of the lateral temporal lobes, early visual cortex, as well as sparse sections of the prefrontal cortex (PFC) and medial frontal lobe. The yellow cluster is located in four distinct areas associated with the default mode network: the PFC, the precuneus, the angular gyrus, and the lateral temporal cortex. Finally, the blue cluster spans large sections of the frontal and parietal lobes, as well as more sporadic areas in the anterior and lateral temporal lobes. Since the 0th-order basis vector is a constant term, and the associated loadings are noninterpretable, we also repeated the clustering procedure excluding them from the analysis, but found no changes in any of the clusters. This is expected, as the data were normalized and thus the loadings on the 0th-order basis vector were close to zero for all voxels.

### Comparison to Functional Connectivity

In order to facilitate the comparison with traditional analyses of the interactions between brain areas, we additionally calculated the functional connectivity between the FFA and all gray matter voxels by taking the correlation of activity in the FFA with the activity in each individual voxel. After averaging the correlation coefficients across all subjects, we were able to map the average functional connectivity of the FFA across the brain. Using a functional connectivity analysis, we were able to determine that the areas showing the strongest correlation with activity in the FFA were the ventral and dorsal temporal lobe, as well as the visual cortex (see Figure 2D). These results highlight a matching set of regions to those identified by the clustering of the linear functional coordinates (Figure 2D).

### Distinct Clusters Are Associated With Unique Functional Relations to the FFA

**Figure 3.**
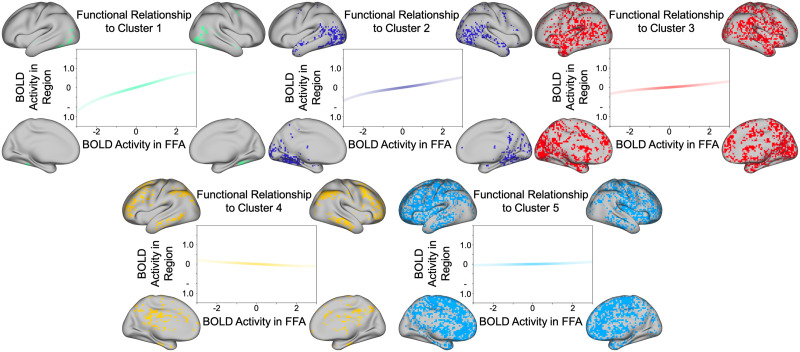
*K*-means clustered voxels. Each plot represents the relationship between (*z*-scored) activity in the FFA (seed region) and the (*z*-scored) activity in the highlighted region. Because the coordinate loadings are defined with respect to the normal probability density function (see formula 11), the intensity of the line color is weighted by the normal probability density function in order to illustrate how the confidence in the prediction of estimated activity varies as activity in the seed region varies. The strongest positive relationship is observed in ventral and lateral posterior temporal regions (green cluster). Note a negative relationship with a cluster of regions in the vicinity of the default mode network (yellow cluster).

One of the central advantages of the functional coordinate analysis is an enhanced interpretability of nonlinear dependencies. Because the nonlinear components of the estimated function are quantified as loadings on basis vectors, we can calculate the interaction between two regions as a weighted sum of the normalized Hermite polynomials. To this end, we can observe the unique computational relationship that defines a given cluster, by taking the loadings from the *k*-means defined cluster center (see Figure 3). After segmenting the gray matter voxels using *k*-means clustering, we identified five distinct clusters with centers at: [4.78*e* − 01, −3.34*e* − 02, 2.23*e* − 02, −2.87*e −* 02], [2.61*e* − 01, −9.47*e* − 03, 2.43*e* − 02, −1.30*e −* 02], [1.35*e* − 01, −6.60*e* − 04, 1.51*e* − 02, −4.85*e* − 03], [−8.28*e* − 02, 8.20*e* − 03, −7.01*e* − 05, 6.32*e −* 03], [3.40*e* − 02, 6.56*e* − 03, 5.40*e* − 03, 1.46*e* − 03]. For each cluster, we calculated the defining function using the respective central functional coordinates as the loadings on the first- through fourth-order basis vectors.

### Clustering Solutions Show Anatomical Consistency With the Increase in the Number of Clusters

**Figure 4.**
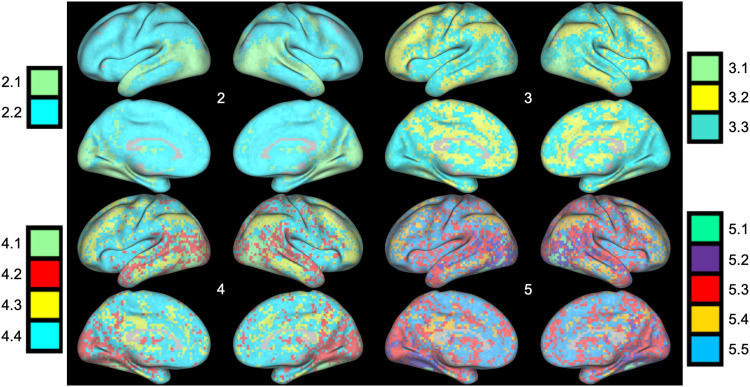
*K*-means clustering of voxels based on five-dimensional functional coordinates, for values of *K* ranging from two to five clusters. As the number of clusters increases, the spatial layout of the new clusters provide a more detailed parcellation of the cortex, but highlight a strikingly similar set of regions.

In order to test the impact of selecting different *k* values on the spatial layout of the resulting clusters, we reran the *k*-means clustering using *k* values ranging from 2 to 5 (see Figure 4). Importantly, we found that the clustering solutions were consistent in their groupings of key areas across the inferior and superior temporal lobe, the visual cortex, as well as the PFC. This consistent anatomical grouping across clustering solutions suggests that our findings are robust across clustering solutions and not dependent on the selection of a distinct number of clusters.

### Coordinates Along Dimensions of the Hilbert Space Reveal the Distribution of Nonlinearities Across Cortex

**Figure 5.**
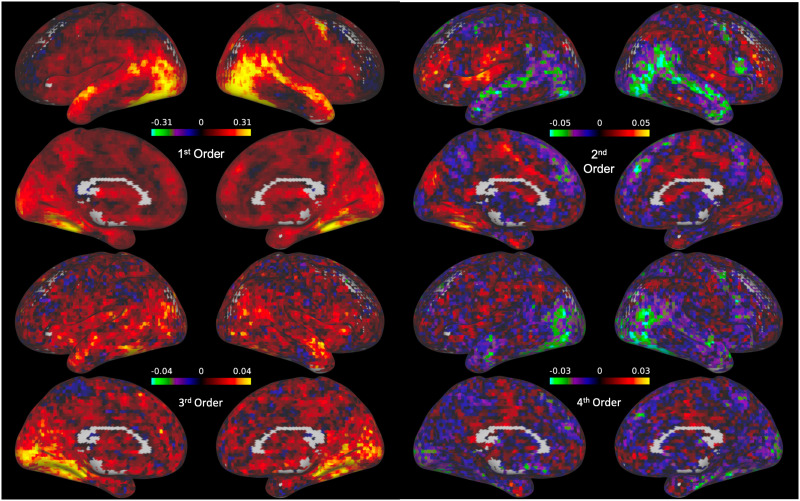
Whole-brain maps of the coefficient estimates for the Hermite polynomials in the basis set (excluding the constant 0th-order polynomial, which captures the baseline signal and thus is not interpretable). Each polynomial in the basis set is associated with a unique cortical map of coefficients. The magnitude of the loadings on the second-order and fourth-order basis vectors are largest in the superior temporal lobe and medial frontal lobe, as well as the face selective visual cortex.

In order to observe the distribution of nonlinear interactions across the cortex, we next plotted the loadings for each of the individual basis vectors for each voxel (see Figure 5) for the loadings on the first basis vector). Interestingly, while the loadings for the linear basis vectors were highest in the regions surrounding the face selective cortical regions and dorsal temporal lobe (Figure 5, first order), nonlinear loadings, particularly in in the second and fourth order basis vectors, were highest in frontal and medial regions, with negative loadings in the face selective cortex and lateral temporal regions. It is worth noting that the magnitude of the basis vector loading reflects the contribution of that basis vector to the overall relationship between the given voxel and the FFA. For this reason, larger loadings on nonlinear polynomials reveal stronger nonlinear components within the given interaction.

**Figure 6.**
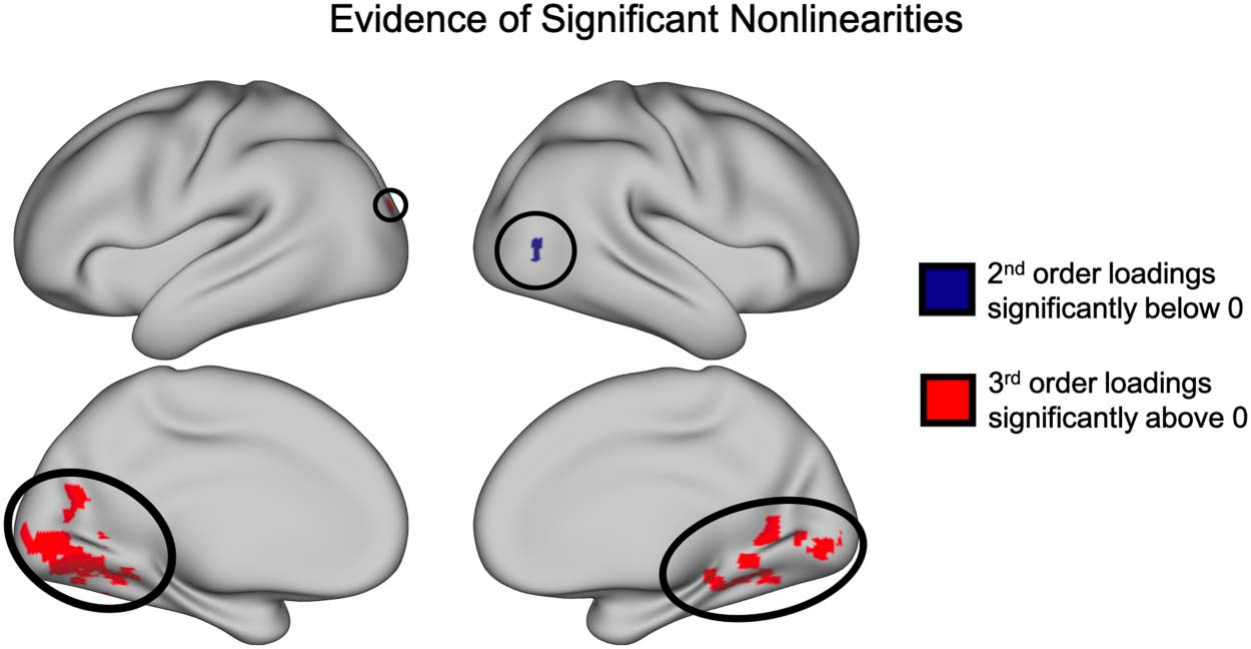
Significant clusters of nonzero loadings (SnPM *t*(13) ≥ 5.59, *p* < 0.05 FWE corrected). Nonparametric pseudo *t* tests revealed a significant cluster of voxels with negative loadings on the second-order basis vector as well as a collection of clusters in the medial occipital lobe and ventral temporal lobe with positive loadings on the third-order basis vector.

To further test the significance of these nonlinear interactions across the cortex, we performed across-subject one-tailed nonparametric pseudo *t* tests using SnPM to highlight any clusters of voxels with significantly nonzero loadings on each of the nonlinear basis vectors (i.e., the second-, third-, and fourth-order basis vectors; Figure 6). These tests revealed one cluster of voxels in area V5 with significantly negative loadings on the second-order basis vector (cluster threshold *p* = 0.0001, *p* (FWE corrected) < .025; see Supporting Information section “Variation in the cluster forming threshold” for additional analyses). Additionally, we found 24 clusters in the early visual cortex and ventral temporal lobe with positive loadings on the third-order basis vector (cluster threshold *p* = .0001; all *p* values (FWE corrected) < .025). We found no significant clusters with nonzero loadings on the fourth-order basis vector.

## DISCUSSION

### Functional Coordinates: A Novel Method to Study Neural Interactions

The major contribution of this research is a novel method to investigate nonlinear interactions between brain regions: “functional coordinates.” Understanding the complexity of interregional connectivity is an important objective within the landscape of current neuroscience research (Anzellotti & Coutanche, 2018; Anzellotti et al., 2017b; Stephan et al., 2008). Importantly, functional coordinates allow for a balance between the interpretability of linear models of connectivity (Friston et al., 1994) and the increased explanatory power and biological plausibility of nonlinear models (Anzellotti et al., 2017b). Moreover, our method offers the ability to characterize *how* the responses in different cortical regions are related, and not just *whether* they are related.

Here, we demonstrated the capacity of the functional coordinate model to capture various types of nonlinearities using synthetic data. Importantly, functional coordinates were able to identify and distinguish between complex nonlinear relationships between data including symmetric (U-shaped) functions, which are particularly difficult to identify using only linear methods. This success on artificial data is an important indicator of the model’s potential to identify the existing nonlinear interactions in real functional data.

Common methods used to study cortical networks rely on linear tools that are incapable of capturing the wide array of potential interactions between brain regions. Functional coordinates can capture the same linear interactions as methods like functional connectivity (Biswal et al., 1995; Rogers et al., 2007). Indeed, the results of our functional connectivity analysis illustrated a high degree of overlap with the clusters defined by the linear functional coordinates. Importantly, however, adding a greater number of functional coordinates captures a greater degree of variability in the statistical dependence across the cortex and allows for an increased level of understanding of the functional relationship to the FFA. Moreover, using the loadings from the nonlinear basis vectors, it is additionally possible to extract the *nonlinear* components of a given relationship.

While the current study used functional coordinates to examine neural interactions of participants during naturalistic movie watching, they can also be used to investigate statistical dependence in resting state data. In addition, this method might be particularly useful for examining task-evoked interactions between areas of interest. For example, in order to detect brain areas that might respond to the emotional arousal of an image regardless of its positive/negative affect, one could perform a searchlight analysis for a region with a U-shaped relationship with regions that respond specifically to the emotional valence of an image. In this way, functional coordinates could play a key role in elucidating complex cortical interactions related to behavior. Some methods capable of capturing nonlinear interactions do exist (Lizier et al., 2011; Wang et al., 2015); however, they often focus on quantifying the *strength* of statistical dependence, rather than studying the *type* of dependence. Importantly, the responses in different target brain regions could be predicted equally well by a given predictor region, yet at the same time, be related to the predictor region via different functions. Functional coordinates are able to quantify the contribution of each nonlinear component of the overall functional relationship, allowing (unlike mutual information) for the explicit definition of the function as a whole (see Figure 3).

One assumption when using functional coordinates is an implied directionality when calculating the nonlinear transformations between brain regions. Importantly, this does not preclude exploratory research into the nonlinear interactions between two brain regions without an explicit hypothesis of which regional activity will be nonlinearly transformed. On the contrary, in this case, calculating functional coordinates bidirectionally may generate novel insight into the reciprocal interactions between these brain areas.

In some respects, functional coordinates are related to estimating a polynomial fit of the interaction between two brain regions and using the vectors of coefficient estimates to characterize the interaction (to our knowledge, this approach has not been proposed in the previous literature). However, functional coordinates offer a key advantage over using the coefficients from a polynomial fit. This is because in a polynomial fit, including higher order nonlinear terms would also lead to changes in the coefficient estimates for the lower order polynomials, making it difficult to compare the results across different studies that used models of different order. By contrast, due to the orthogonality of Hermite polynomials, functional coordinates are such that the estimates of the coefficients of lower order basis functions do not change when the model is expanded with the addition of higher order basis functions.

Moreover, functional coordinates can also be scaled up to take multidimensional patterns of activity as input, much like existing methods such as MVPD (Anzellotti et al., 2017a) and Informational Connectivity (Coutanche & Thompson-Schill, 2013), through the use of multivariate Hermite polynomials. This advantage allows for an examination of the rich high-dimensional nature of neural data and provides the opportunity to incorporate a wider array of neural response patterns into the study of nonlinear interactions between brain regions.

One potential future adaptation of functional coordinates might be to expand their use in order to examine the relationship between neural activity across individuals. Functional coordinates could be leveraged to contribute to the existing work on intersubject correlation (Hasson et al., 2004), which examines the correlation of brain activity between two subjects. Intersubject correlation provides a method to study a proxy measure of how much information is encoded in a given voxel across multiple brains (Nastase et al., 2019); by calculating a set of functional coordinates to characterize the interaction of neural activity across individuals, one might gain further insight into the relationship between the amount of information encoded across these distinct brains.

### Evidence of Nonlinear Interactions Between Brain Regions

In this work, we also demonstrate the existence of distinct nonlinear interactions that characterize the relationships between the FFA and other regions across the brain. After first defining a set of functional coordinates to describe the relationship of the average FFA activity with the activity in each other gray matter voxel, we were able to cluster the functional coordinates across all voxels to identify five distinct sets of voxels with different functional interactions (see Figures 2C and 3). Importantly, these clusters differ from those identified with a linear model of connectivity (see Figure 2B) highlighting the increased ability to discriminate between voxels based on their relationship to the seed region.

Upon mapping out the functions associated with each cluster (Figure 3), we were able to determine the functional forms of the relationships between activity in the FFA and activity across the rest of the brain. A key feature of these relationships is their nonlinear components. While the loadings on the linear basis vectors are, indeed, still the predominant component in the defined clusters, the loadings on the nonlinear component are also important for differentiating the patterns of connectivity across voxels. This is apparent in the increase in the optimal number of clusters calculated for the five-dimensional functional coordinates (five clusters) as compared to the clusters for the linear loadings only (two clusters). Noticeably, voxels surrounding face selective regions and those in the anterior temporal lobe were grouped into a single cluster when only including the linear component; in contrast, when clustering across all five basis vectors, this same group of voxels was, instead, classified into separate, functionally distinct clusters. It is worth noting, that although we would expect the optimal number of clusters to increase with additional basis vectors (because they provide additional information regarding higher order nonlinearities), the number of clusters is *not* necessarily equal to the number of Hermite polynomials used. For example, clustering across only the linear basis vector resulted in an optimal solution with two clusters.

Moreover, we report additional evidence for the importance of discovered nonlinearites in the significant clusters of positive loadings on the third-order basis function and negative loadings on the second-order basis function (Figure 6). These clusters demonstrate specific areas in both early visual cortex as well as downstream visual processing regions with which the FFA has significant nonlinear interactions. In a follow-up analyses (see Supporting Information, Regressed Nonlinear Loadings) we regressed out the linear basis vector loadings from those of the nonlinear basis vectors in order to discover any nonlinearities whose spatial distribution across cortical voxels is decoupled from that of linear interactions. With this analysis, we found two additional clusters that were trending toward significance in the posterior superior temporal sulcus (pSTS) and the anterior temporal lobe (Supporting Information, Figure S13), two regions previously implicated in face perception (Anzellotti & Caramazza, 2017; Rajimehr et al., 2009).

These clusters suggest the possible presence of very localized, anatomically specific nonlinear interactions between FFA and other regions in the face network, but additional studies will be needed to evaluate the robustness of this finding.

### Potential Pitfalls in the Search for Nonlinearities

Although, in this work, we present evidence of significant nonlinear interactions between brain regions, it is important to note that the linear components of these interactions were still an order of magnitude larger than the nonlinear loadings. Moreover, while we did find significant clusters of nonzero nonlinearities, these regions were particularly small and highly localized.

This development in our understanding of nonlinear cortical interactions is intriguing, in part, because existing computational and biological evidence suggests that nonlinearities are a key part of neural information processing. Indeed, at the level of individual neurons, dendrites perform nonlinear integration of signals (Lafourcade et al., 2022; Tran-Van-Minh et al., 2015; Xu et al., 2012). One recent study found that approximating the input/output relationship of a single pyramidal neuron requires using a deep recurrent neural network with five to eight layers (Beniaguev et al., 2021). Other computational evidence has shown that nonlinearities are a key feature of models (like deep artificial neural networks) that approximate human behavior (Yamins et al., 2014). Although previous research has not been able to identify substantial nonlinear interactions (Hlinka et al., 2011), the advancement of fMRI preprocessing and denoising techniques in the past 15 years could have revealed more evidence of nonlinearities. The results of the current study are therefore surprising in two key ways: first, they do not demonstrate widespread nonlinear cortical interactions, in apparent contrast with the existing biological and theoretical evidence. Second, however, we do detect evidence of *some* nonlinearities, which suggests that there is promise for future research studying the nonlinear interactions between brain regions in fMRI data. This pattern of results raises an important question for research moving forward: why do nonlinear interactions not account for a greater proportion of the variance in the interactions between brain regions?

The first potential explanation of the dearth of significant nonlinear interactions is that our model is incapable of identifying the full extent of the existing nonlinearities in the relationships between BOLD activity in different regions. However, our simulation data show that the method is able to accurately capture nonlinearities, at least within the set of cases we tested. In addition, it is important to note that we *do* report evidence of significant nonlinearities between the FFA and early visual cortex, V5, and medial temporal areas (see Results; Coordinates Along Dimensions of the Hilbert Space Reveal the Distribution of Nonlinearities Across Cortex section). This suggests that our model is *capable* of capturing existing nonlinear interactions, but that the magnitude of these interactions in the fMRI data we analyzed is small. Additionally, our current model has outperformed previous models attempting to capture the nonlinear interactions with the FFA within this same dataset (Poskanzer et al., 2022). In sum, the small effect size of the nonlinearities appears to be a feature of the data, and not a consequence of the chosen model.

Another possibility that could explain the difficulty in discovering nonlinear interactions is that fMRI data, in particular, is not suitable to measure them. Previous work in this area has demonstrated that contributions of nonlinear models of functional connectivity in resting-state fMRI have been relatively minor when compared to their linear counterparts (Hlinka et al., 2011). This result could be, in part, due to a number of underlying limitations of fMRI data, including spatial resolution, temporal resolution, and hemodynamic smoothing. A further difficulty in the effort to examine nonlinear interactions in fMRI data may be the difficulty in separating out the signal of interest from nonneural sources of fluctuations in the data. Although, CompCor has been shown to effectively remove signals of no interest, future denoising techniques may be better equipped to reveal the underlying complexities of neural activity.

When considering the mechanics of connectivity at a neural level, it is entirely possible that the nonlinear dynamics that we might expect to see are evident at a spatial scale that is too fine-grained for the millimeter resolution of an MRI scanner. At the cellular level, neurons are able to nonlinearly integrate signals from multiple synapses (Beniaguev et al., 2021; Xu et al., 2012). In contrast, BOLD signal at the voxel level reflects the activity of hundreds of thousands of neurons. In this way, spatial smoothing might “blur” the complex interactions occurring at the neuronal level and, thus, minimize the underlying nonlinear interactions. In a similar fashion to the spatial smoothing, fMRI data also suffers from temporal smoothing: our data might not be sampled at a high enough rate to map any nonlinear interactions that occur on the order of milliseconds. However, the averaging of a large number of nonlinear functions does not generally produce a linear function. If the loss of nonlinear information in fMRI signal is due to averaging over space and time, this would suggest that the amount and type of nonlinear interactions between neurons in different brain regions are such that their average is approximately linear.

Finally, another complicating factor of fMRI data is smoothing as the result of the hemodynamic function. Neural activity is nonlinearly related to the BOLD signal (de Zwart et al., 2009). This proxy measurement of the underlying activity of cortical neurons can serve, in this case, as an additional smoothing function that might decrease the detectability of any nonlinear neural interactions.

Despite the possibility that the BOLD signal may not be the optimal tool for the study of the fine-grained nonlinear interactions between brain areas, it is also distinctly possible that it not the *quality* but the *quantity* of data that is at issue. Hlinka et al. (2011) only reported finding “subtle” nonlinear effects by “testing across many pairs or even across many sessions.” In this study, we also found “subtle” yet significant nonlinear interactions by testing across a series of subjects. One potential avenue for future research might be to examine datasets comprising extensive scans of small numbers of participants (Allen et al., 2022). Using many runs of single subject data and avoiding the averaging across subjects could increase the chances of identifying more subtle effects.

Although it is not currently clear why the scale of nonlinear interactions between brain regions is comparatively much smaller than the scale of linear interactions, we believe that solving this problem is essential for progress in the field of computational neuroscience. It is clear that nonlinear computations are crucial for models that approach human behavior on cognitive tasks (Yang et al., 2019) and for models that capture the behavior of biological neurons (Beniaguev et al., 2021). Applying methods such as functional coordinates to very large fMRI datasets, or to datasets acquired with more direct measures of neural activity (i.e., neuropixels; Steinmetz et al., 2018), is likely to shed new light onto the neural bases of cognitive computations.

## ACKNOWLEDGMENTS

This work was funded by the Department of Psychology and Neuroscience at Boston College.

## SUPPORTING INFORMATION

Supporting information for this article is available at https://doi.org/10.1162/netn_a_00264.

## AUTHOR CONTRIBUTIONS

Craig Poskanzer: Data curation; Formal analysis; Investigation; Methodology; Writing – original draft; Writing – review & editing. Stefano Anzellotti: Conceptualization; Funding acquisition; Methodology; Supervision; Writing – review & editing.

## FUNDING INFORMATION

Stefano Anzellotti, Simons Foundation Autism Research Initiative (https://dx.doi.org/10.13039/100014370), Award ID: 614379.

## Supplementary Material



## TECHNICAL TERMS

Functional connectivity: The correlation between the neural responses in two brain regions.
Function estimation: Identifying a function that approximates another function.
Hilbert space: A vector space with an inner product that defines a distance with respect to which the vector space is a complete metric space.
Basis functions: A set of functions in a function space such that any function in the space can be expressed as a linear combination of the functions in that set.
Dynamic causal modeling: A technique used to infer the strength and directions of interregional interactions and their fluctuations.
Granger causality: A method that determines the influence of *x* on *y* by using past *x*-values to predict future *y*-values.
Mutual information: A measurement of how much information one variable gives about another.
Multivariate pattern dependence: A technique to study the multivariate interactions between brain areas.
Hermite polynomials: A group of polynomials that are a basis set for a Hilbert space; orthogonal with respect to the normal distribution.
Seed region: A region of interest chosen in order to study its interactions with the rest of the brain.
