# Supplementary material for: Functional coordinates: Modeling interactions between brain regions as points in a function space
Source: Netw Neurosci. 2022 Oct 1;6(4):1296–315. doi: 10.1162/netn_a_00264 (PMC11117108; doi:10.1162/netn_a_00264)
Supplement: Supplementary file 1 [file netn-6-4-1296-s001.pdf]

Poskanzer, C. & Anzellotti, S. (2022). Supporting information for "Functional coordinates: Modeling interactions between brain regions as points in a function space." *Network Neuroscience*. Advance publication. https://doi.org/10.1162/netn_a_00264

Supporting Information

The first five normalized Hermite polynomials

$H_0 = 1/\sqrt{\sqrt{2\pi}0!}$
$H_1 = x/\sqrt{\sqrt{2\pi}1!}$
$H_2 = (x^2 - 1)/\sqrt{\sqrt{2\pi}2!}$
$H_3 = (x^3 - 3x)/\sqrt{\sqrt{2\pi}3!}$
$H_4 = (x^4 - 6x^2 + 3)/\sqrt{\sqrt{2\pi}4!}$

Normality of the Data

See Figure 7

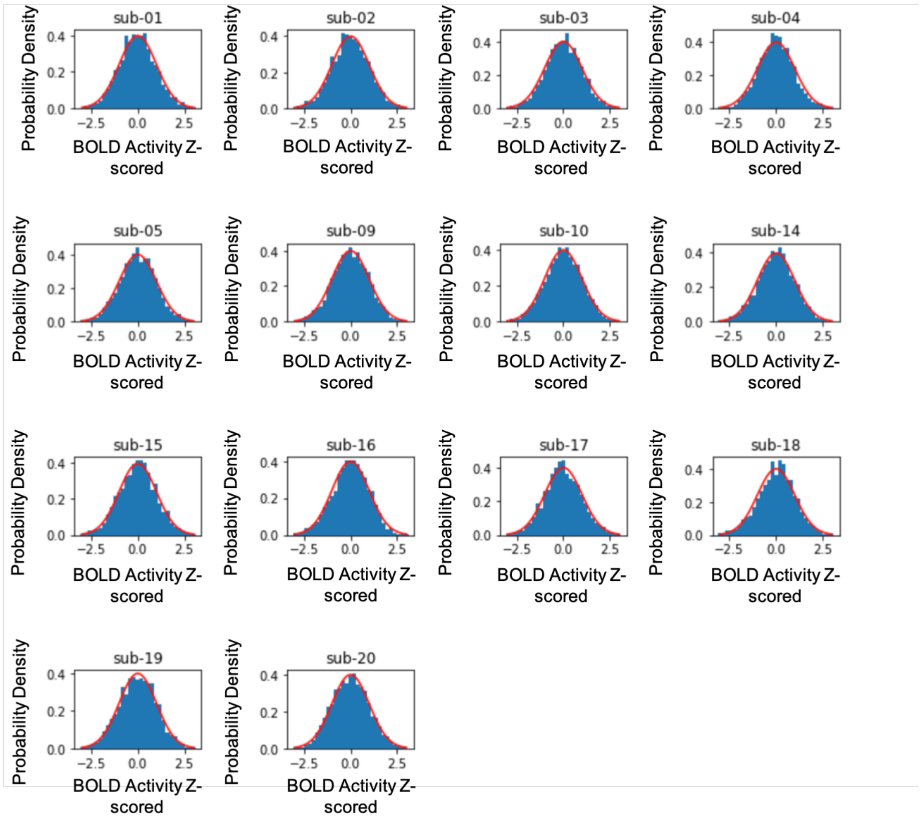

Figure 7: The denoised functional data for each individual subject were Z-scored and plotted as probability density histograms (blue) with the probability density function of the normal distribution (red) superimposed.

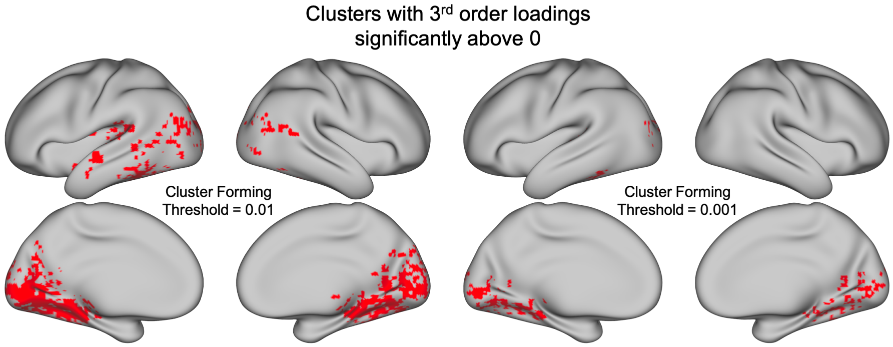

Figure 8: Clusters of voxels with loadings on the 3rd order basis vector that were significantly above 0. Using a cluster forming threshold of $p = 0.01$ (left) and $p = 0.001$ (right) we found broader significant clusters of voxels that had a loadings greater than zero on the nonlinear, 3rd order basis vector. We no longer found any significant clusters of non-zero loadings on the 2nd order basis vector.

Location of the FFA

Average MNI Coordinates for FFA	
Right Hemisphere	Left Hemisphere
(42.9, -47.9, -18.5)	(-43.2, -49.8, -18.3)

Variations in the cluster forming threshold

When performing tests of the significance of the nonlinear loadings, we used a cluster forming threshold of $p = 0.0001$. To further examine the how variations in the cluster forming threshold may impact the the spread of significant clusters of nonlinear loadings, we ran two additional across-subject non-parametric pseudo T-tests on the 2nd and 3rd order loadings using a cluster forming threshold of $p = 0.01$ and $p = 0.001$. In both cases, we no longer found any significant clusters of negative 2nd order loadings. Using a cluster forming threshold of $p = 0.01$, we found a single cluster across the visual cortex and ventral temporal lobe of positive 3rd order loadings (Figure 8). Finally, using a cluster forming threshold of $p = 0.001$, we found 4 clusters across the visual cortex and ventral temporal lobe with 3rd order loadings significantly greater than 0 (Figure 8).

Explained variance of each basis function

Another method to compare how well the estimated functional coordinates fit the data is to calculate the variance explained in the data by each individual coordinate. Here, for each subject, we predicted the activity in all voxels using each polynomial separately. For each voxel, v, we used the estimated coordinates

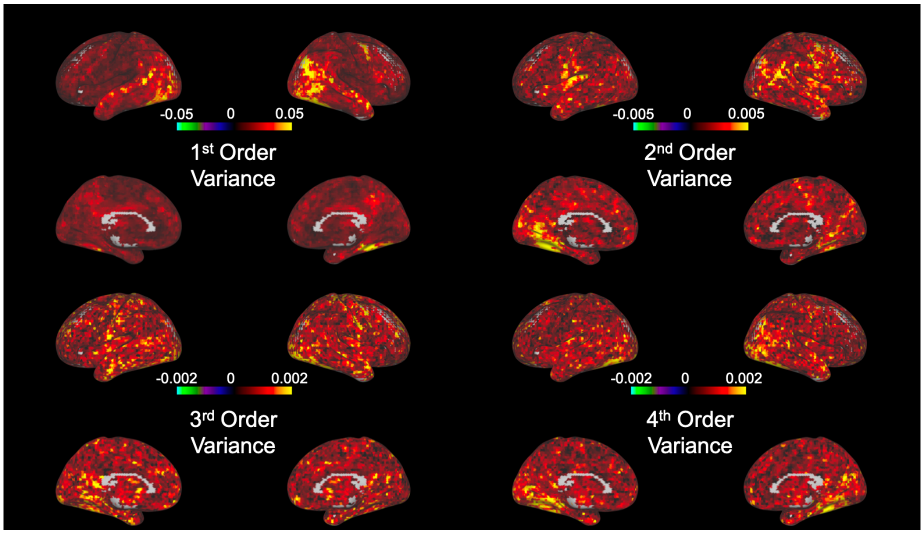

Figure 9: For each polynomial, the variance of the loadings on that coordinate across all subjects is plotted for each voxel. This illustrates how consistent the loading values are across subjects for each voxel.

as loadings onto the respective basis functions in order to predict that voxel's activity as a function of activity in the FFA, such that

$$EstimatedActivity_v = c_i H_i(Activity_F FA), \tag{14}$$

where c_i is the loading for the ith order basis function and H_i is the ith order Hermite polynomial. We then used this prediction to calculate the variance explained in the true activity for that voxel. The procedure was completed for all voxels in all subjects and averaged across subjects (Figure 10). Explained variance for each basis function was highest in the areas where loadings had the largest magnitudes.

Uncertainty of the Coordinates

To capture the uncertainty of the clustering solution for each voxel, we calculated the proportion of subjects who shared the cluster assignment for that specific voxel (as described in Methods, Data Analysis). These proportions were used to adjust the color saturation of each voxel in Figure 2, B and C. We have additionally plotted the proportion of agreement for each voxel in Figure 11.

In order to capture the uncertainty of the estimated loadings on each polynomial, we ran an 8 fold cross validation. For each fold of the cross validation, one of the 8 functional runs was removed from the data used to estimate the loadings of each coordinate. This procedure was performed 8 times such that each run was removed once. This means that for each subject, the functional

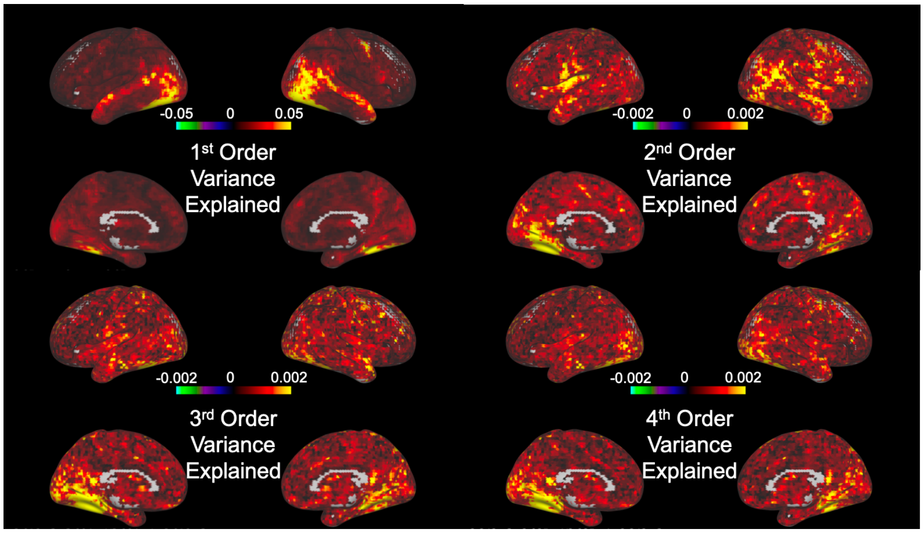

Figure 10: The explained variance in the activity for each voxel by each coordinate-weighted basis function (averaged across subjects). We compared the variance explained by each basis function by using the functional coordinates to estimate the activity in each voxel in reference to the true activity of that voxel.

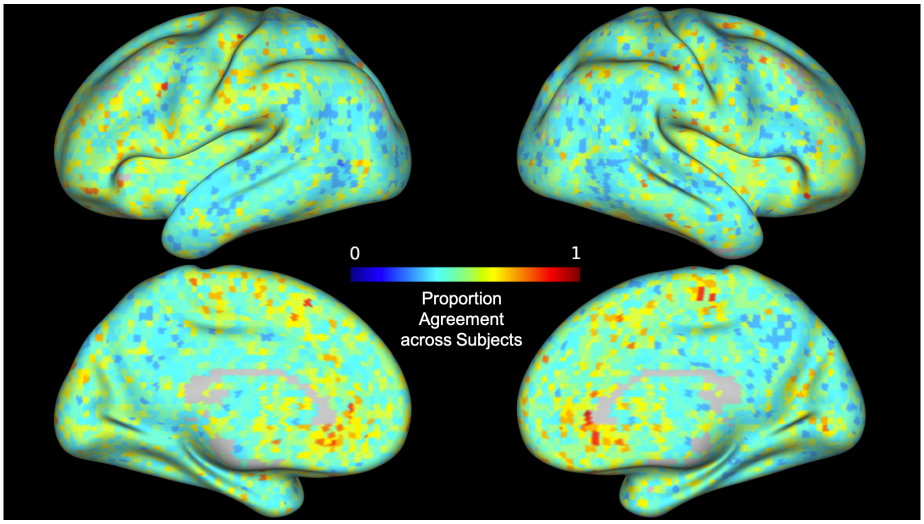

Figure 11: The final clustering solution produced a cluster assignment for each voxel in each subject. We then took the most common cluster assignment across subjects and assigned that voxel to that cluster. Plotted here, are the proportions of subjects who shared that cluster assignment.

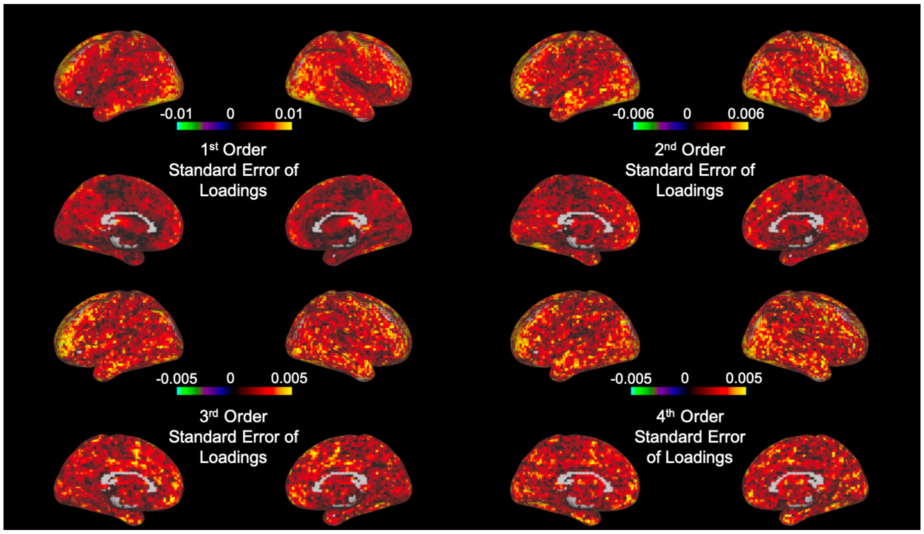

Figure 12: Coordinates for each basis function (not including the 0th order) were estimated using an 8 fold cross validation procedure. For each voxel, the standard error across the estimated coordinates was calculated. Plotted above are the average standard errors of the loadings for the 1st, 2nd, 3rd, and 4th basis functions for all voxels.

coordinates were estimated 8 separate times on 8 different combinations of the functional data. Next, for each coordinate, the standard error across all estimated loadings was calculated and then averaged across all subjects (Figure 12).

Regressed Nonlinear Loadings

In addition to our analysis of the significance of the loadings on the nonlinear basis functions, we further probed the significance of the nonlinear loadings after regressing out the loadings from the linear basis function. To regress out the loadings from the linear basis function, we take the loadings for a higher order polynomial as a vector y. We also extract the loadings for the first order (linear) polynomial as another vector x. We then estimate the model $y = ax+b+epsilon$. Finally, we keep and visualize the residual epsilon. We performed this analysis in order to to observe any nonlinearities that were uncorrelated with the linear interactions. We found 2 clusters of loadings on the 2nd order basis function that trended towards being significantly below zero: one in the pSTS ($p = 0.04$, FWE corrected one-tailed non-parametric pseudo t-test as implemented in SnPM), and one in the anterior temporal lobe ($p = 0.04$, FWE corrected one-tailed t-test) (Figure 13).

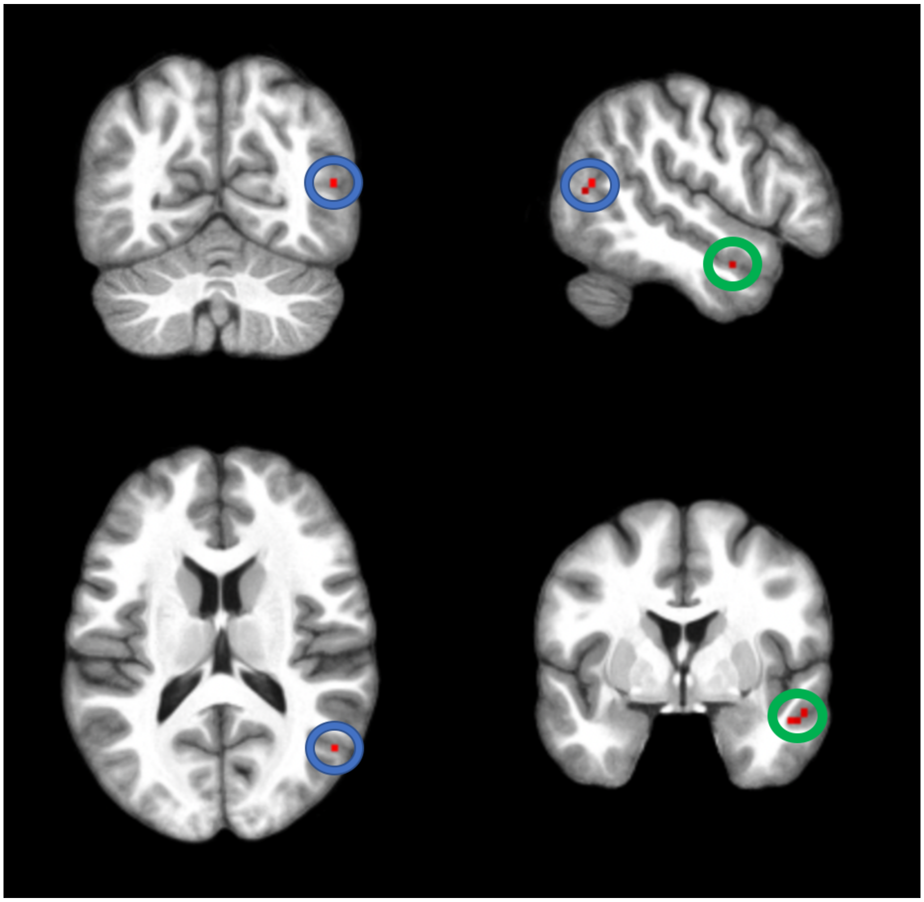

Figure 13: Clusters of below-zero loadings on the regressed 2nd order basis function.
